# Benign mixed epithelial stromal tumor of the renal pelvis with exophytic growth: case report

**DOI:** 10.1186/1477-7800-2-18

**Published:** 2005-09-09

**Authors:** Yuvaraja B Thyavihally, Hemant B Tongaonkar, Sangeeta B Desai

**Affiliations:** 1 Department of Urologic oncology, Tata Memorial Hospital, Dr E. Borges Road, Parel, Mumbai-40012, India; 2 Department of Pathology, Tata Memorial Hospital, Dr E. Borges Road, Parel, Mumbai-40012, India

## Abstract

**Background:**

Mixed epithelial and stromal tumor (MEST) is a distinctive benign composite neoplasm of the kidney predominantly seen in females mostly in the perimenopausal period. Although these tumors are known to arise from renal pelvis, our case was distinct in that it had no intrapelvic component growing in exophytic fashion.

**Case report:**

A 35 year old female patient presented to us with vague abdominal pain. She had undergone excision of bilateral ovarian cystic masses for cystic teratoma twelve years earlier. A computed tomography scan of abdomen and pelvis showed a 9 × 7 cm uniformly solid mass with poor contrast enhancement situated in the inferomedial aspect of the left kidney. On exploration, the mass was arising from the inferior and anterior aspect of left renal pelvis, and was attached to it with a narrow pedicle. There was no adherence or attachment to the renal parenchyma. The mass was excised preserving the kidney. Microscopically, the tumor was composed of large collagenized areas containing bundles of spindle cells and several 'microcysts' lined by cuboidal epithelium suggestive of a benign mixed epithelial stromal tumor.

**Discussion:**

Mixed epithelial tumors usually present in perimenopausal women as a partially cystic mass. Tumors are composed of irregular mixtures of cystic and solid areas, glands with variable complexity and distribution and the stromal component is characterized by a spindle cell proliferation. Commonly, it arises from the renal parenchyma and pelvis and nephrectomy is advocated to manage these tumors.

**Conclusion:**

MEST is a distinctive benign tumor of the kidney that should be distinguished from other renal neoplasms. MEST arising from the renal pelvis and growing exophytically is a rare entity. The overall prognosis is favorable.

## Background

The mixed epithelial and stromal tumor (MEST) is a distinctive benign tumor of the kidney that should be distinguished from other renal neoplasms. The name MEST was suggested by Michal and Syrucek in 1998 [1]. The tumor is characterized morphologically by a mixture of solid and cystic areas which consist of a biphasic proliferation of glands admixed with solid areas of spindle cells with variable cellularity and growth patterns [2,3]. Most are benign although malignant variety has been described.

We report an unusual case of benign mixed epithelial stromal tumor arising from the renal pelvis and growing in an exophytic fashion. This was treated by excising the mass without nephrectomy.

## Case presentation

**Figure 1:**
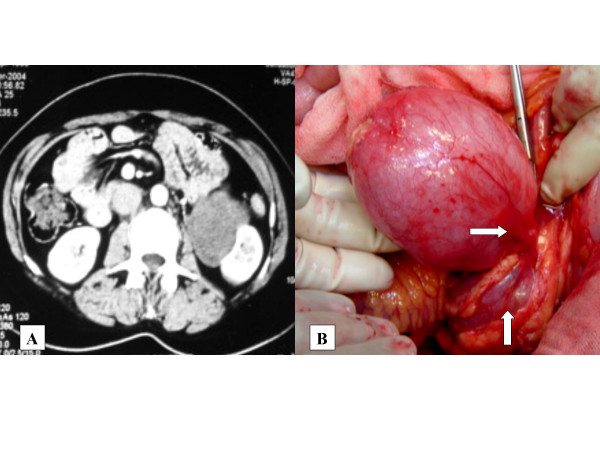
A. CT scan showing homogenous mass located at medial aspect of the left kidney and compressing it with minimal contrast enhancement. B. Intraoperative picture demonstrating the mass which is free from renal parenchyma (vertical arrow) and attached to renal pelvis with a narrow pedicle (horizontal arrow)

A 35 year old unmarried female patient presented to us with abdominal pain. On examination, a vague mass was palpable in the left lumbar region. The patient had history of excision of bilateral ovarian cystic masses for cystic teratoma twelve years earlier. Computerized tomographic scan of the abdomen and pelvis showed a 9 × 7 cm uniformly solid mass with poor contrast enhancement situated in the inferomedial aspect of the left kidney [Fig 1A]. It was difficult to determine whether the mass was arising from renal parenchyma or renal pelvis. There were no retroperitoneal lymphnodes. Urine cytology, cystoscopy and retrograde ureterography were normal. There was no evidence of distant metastases. The patient also had a pelvic adnexal mass. On abdominal exploration, there was a well defined mass arising from the inferior and anterior aspect of the left renal pelvis, and attached to it with a narrow pedicle. There was no adherence or attachment to the renal parenchyma [Fig 1B]. The renal pelvis was opened to remove it completely. There was no intrapelvic growth. The part of the renal pelvis (about 1.5 cm) from which the tumor was arising was excised. Frozen section revealed a benign nature. The renal pelvis was closed after inserting a double J stent into left ureter. The left adnexal mass was excised with preservation of uterus and other ovary.

On pathological examination, the tumor was well-defined and capsulated. On cut section it had a firm, whitish solid appearance without areas of necrosis or hemorrhage.

**Figure 2:**
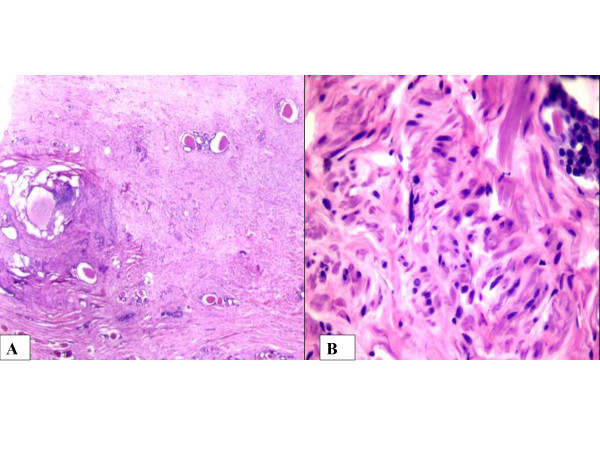
A. The scanner view (×25): B. High power picture (×100) Biphasic tumor with epithelial and stromal component. Stroma: Collagenized areas containing bundles of spindle cells. Epithelial component: Several 'microcysts' lined by cuboidal epithelium.

Microscopically, the tumor was composed of large collagenized areas containing bundles of spindle cells. These areas were interspersed with thick walled, blood vessels, fatty tissue and several 'microcysts' lined by cuboidal epithelium [Fig 2A, B]. Normal renal parenchyma was not seen. Immunohistochemistry showed strong positivity for SMA and desmin in the spindle cell areas. Cytokeratin (CK) and epithelial membrane antigen (EMA) were positive in cells lining the microcysts. Estrogen receptor (ER), Progesterone receptor (PR) and HMB 45 (Human melanoma antibody) were negative. The pelvic adnexal mass was found to be a mature cystic teratoma. After one year of follow up, the patient was well with no adverse effects from the surgery.

## Discussion

The mixed epithelial and stromal tumor is a recently established tumor of the kidney of unknown etiology. It has a well defined capsule and solid and cystic areas, either of which may predominate. The solid parts are homogeneous, yellow-white, and firm and rubbery in some areas; they are fleshy and tan in other areas, corresponding to variable stromal cellularity [1].

The cysts range from millimeters to several centimeters in diameter and may be clustered in separate foci or dispersed in solid areas in a "Swiss cheese" pattern. The solid areas show mesenchymal elements which may be spindle cells or may resemble smooth muscle cells; but do not show atypia or increased mitoses or blastema. The epithelial component of this tumor consists of glands with variable complexity and distribution, and the stromal component is characterized by a spindle cell proliferation with cellularity akin to scar tissue to appearances that are reminiscent of leiomyoma, ovarian stroma or solitary fibrous tumor. The spindle cells of this tumor frequently express estrogen/ progesterone receptors.

A possible cell of origin for these tumors is the primary mesenchymal metanephric blastema that has the capacity for mesenchymal and epithelial differentiation. An alternative theory is based on the distinctive clinical and pathologic features of this lesion that characterize a hormonal mechanism of pathogenesis. It is postulated that a deranged hormonal milieu (perimenopausal changes or therapeutic hormones with unopposed estrogen) induces proliferation of periductal foetal mesenchyme, which in turn drives the growth of the epithelial component [2].

About 50 cases of MEST have been reported in the literature. It usually presents in perimenopausal women as a partially cystic mass and its growth may be influenced by hormones. Most patients present with flank pain, hematuria, or symptoms related to genitourinary infections. About 20% are asymptomatic, and tumors are detected incidentally during investigation for other diseases. MEST occurs in women at a 6:1 ratio over men, at a mean age of 46 years. In one reported series, 7 of 10 women had long histories of treatment with estrogen, in 6 of these following oophorectomy several years earlier [1]. The only male patient had a 7-year history of diethylstilbestrol therapy followed by 4 years of treatment with leuprolide acetate.

Although benign, cases of malignant mixed epithelial and stromal tumors of the kidney have been reported with fatal outcome by Svec et al, Bisceglia and Bacchi [4-6].

MEST is previously reported under different names as 'Adult mesoblastic nephromas' 'Cystic hamartoma of renal pelvis' 'Cystic nephroma'. The tumor frequently contains areas of smooth muscle in which epithelial structures are embedded, hence, some have concluded that it is an adult form of congenital mesoblastic nephroma but this is contentious [7]. MEST of the kidney lacks genetic alterations typical of congenital mesoblastic nephroma, such as ETV6-NTRK3 gene fusion or polyploidy of chromosomes 8, 11 and 17 [8].

Other differential diagnoses of mixed epithelial and stromal tumor include rare biphasic renal tumors with a malignant spindle cell component: cystic embryonal sarcoma and sarcomatous transformation in cystic nephroma, both of which can be distinguished from mixed epithelial and stromal tumors by their overtly malignant stroma with cytologic atypia, mitotic figures and necrosis.

Immunohistochemical stains, such as HMB 45 (frequently positive in angiomyolipoma) and CD34 (a sensitive marker of solitary fibrous tumor), may be helpful in excluding some of these alternative diagnoses.

A series of 22 patients showed that all tumors were arising from renal parenchyma and six having tumor bulging into the renal pelvis [9]. Yamasaki et al reported a case of MEST occurring as a polypoidal growth inside the renal pelvis [10]. Our case is unique in that the tumor was attached to the anterior and outer surfaces of the renal pelvis and growing exophytically without an intrapelvic component and furthermore, was compressing the renal parenchyma without infiltration. The mass could be excised without removal of the kidney.

## Conclusion

In summary, "mixed epithelial and stromal tumor" is a distinctive benign tumor of the kidney that should be distinguished from other renal neoplasms. MEST arising from the renal pelvis and growing exophytically has not reported previously. It is difficult to distinguish between benign or malignant nature on imaging studies. Intra-operative frozen section is necessary to exclude a malignancy and an attempt for a conservative surgery is worthwhile. The overall prognosis is favorable.

## Competing interests

The author(s) declare that they have no competing interests.
